# Benefits and constrains of covalency: the role of loop length in protein stability and ligand binding

**DOI:** 10.1038/s41598-020-76598-x

**Published:** 2020-11-18

**Authors:** Sara Linse, Eva Thulin, Hanna Nilsson, Johannes Stigler

**Affiliations:** 1 Departments of Biophysical Chemistry, Biochemistry and Structural Biology, Lund University, Lund, Sweden; 2 Gene Center, Ludwig-Maximilians-University, 81377 Munich, Germany

**Keywords:** Protein folding, Proteins

## Abstract

Protein folding is governed by non-covalent interactions under the benefits and constraints of the covalent linkage of the backbone chain. In the current work we investigate the influence of loop length variation on the free energies of folding and ligand binding in a small globular single-domain protein containing two EF-hand subdomains—calbindin D_9k_. We introduce a linker extension between the subdomains and vary its length between 1 to 16 glycine residues. We find a close to linear relationship between the linker length and the free energy of folding of the Ca^2+^-free protein. In contrast, the linker length has only a marginal effect on the Ca^2+^ affinity and cooperativity. The variant with a single-glycine extension displays slightly increased Ca^2+^ affinity, suggesting that the slightly extended linker allows optimized packing of the Ca^2+^-bound state. For the extreme case of disconnected subdomains, Ca^2+^ binding becomes coupled to folding and assembly. Still, a high affinity between the EF-hands causes the non-covalent pair to retain a relatively high apparent Ca^2+^ affinity. Our results imply that loop length variation could be an evolutionary option for modulating properties such as protein stability and turnover without compromising the energetics of the specific function of the protein.

## Introduction

A protein domain may be defined as an independent folding unit; if excised from the rest of the protein, or produced separately, it folds into the same globular structure as in the intact protein^1,2^. A protein may consist of only a single domain but many proteins contain multiple domains. This is particularly true for some extracellular proteins, such as von Willebrand Factor or cytoskeletal and muscle proteins, such as filamin or titin, where a large number of domains is connected in the same polypeptide chain^3,4^.

A subdomain—also called supersecondary structure or motif—may be defined as a covalent unit within a domain that is stable only in the context of the parent domain but thermodynamically unstable in isolation. Examples of such subdomains are helix-loop-helix, strand-loop-strand or zinc finger motifs. Subdomains are joined by short or long loops forming ordered or flexible segments of the folded protein domains. The influence of loop length on protein folding and stability has been studied in systems such as the designed four-helix-bundle protein Rop^5,6^, chymotrypsin inhibitor-2^7^, ⍺ spectrin SH3^8^, yeast phosphoglycerate kinase^9^, cytochrome c^10^ or human muscle acylphosphatase^11^. The three dimensional structures of loop-length variants of these proteins are native-like, but the thermodynamic stability of such engineered domains decreases with loop length^5–7,9–11^. Each protein folds via the same general mechanism irrespective of loop length, but the folding rates are lower the longer the linker, while the unfolding rates are unaffected^7^. Crucially, the native structure can still remain unaffected when the chain topology is altered by circular permutation, i.e. linking of N- and C-termini while cutting another loop, albeit at the expense of an altered folding pathway^12^.

These examples illustrate the importance of non-covalent interactions in governing the native fold of a protein. The extreme case of very long linker may approach the situation of disconnected subdomains. Indeed, for many proteins, an intact polypeptide chain is not required for the native fold and function. The first classical example of this property of fragment complementation is ribonuclease which was reconstituted 1958 from two separate polypeptide fragments with retained fold and function^13^. This exercise has been followed by many reports of proteins that are stable enough that their structure and function can be reconstituted through the non-covalent assembly of fragments comprising two or more subdomains^14–18^. The individual subdomains may be more or less unfolded in isolation but their folding is energetically coupled to association with the other subdomain. Fragment complementation illustrates that folding can also occur efficiently regardless of covalent linkage, which implies a large tolerance also to loop length variation between subdomains and secondary structure elements. Protein reconstitution thus also shares many features of the folding-upon-binding reaction for intrinsically unfolded proteins. In a related phenomenon, 3D domain swapping, two protein chains trade subdomains and form dimers in which the native fold is reconstituted twice from the subdomains on the two chains^18–20^. This phenomenon is not limited to dimers; higher order oligomers may also form through domains swapping and the phenomenon may even lead to the formation of gels or extended aggregates via runaway-domains swapping^21,22^.

The EF-hand helix-loop-helix motif, first discovered in parvalbumin^23^, is a prototypical subdomain with over 1000 examples known^24^. While each EF-hand may coordinate one calcium ion between residues in the loop region^25^, the binding free energy is dominated by the entropic gain from releasing the water molecules that were coordinating the calcium ion in the unbound state^26^. Protein domains of troponin C^15^, calmodulin^27^, calbindin D_9k_^16,28^, calbindin D_28k_^29,30^, calrethinin^31^ and sarcoplasmic Ca^2+^-binding protein^32^ have been reconstituted from fragments containing one or more EF-hand subdomains. These studies have established that isolated EF-hand fragments may fold upon Ca^2+^ binding, in which case they form homodimers^15,16^ or higher order assemblies^33^ to avoid the entropic penalty of exposing a large hydrophobic surface to surrounding water. Upon mixing two of EF-hand homodimers from the same protein, they spontaneously and rapidly redistribute to form the heterodimer, with very high yield as the heterodimer is thermodynamically very much favored in the presence of Ca^2+^^15,16,34^. Studies of calbindin D_9k_ show that disulfide linkage within each homodimer slows down the reconstitution process to the minutes time scale, as disulfide exchange becomes rate-limiting for redistribution to heterodimer^28^. The high preference for EF-hand hetero- vs homodimer has been rationalized in terms of optimized packing of hydrophobic side chains in the heterodimer and electrostatic repulsion in one homodimer, using EF-hand phage display^35^. EF-hand reconstitution has moreover been used as a purification strategy for membrane proteins^36^, as a tool to study the role of hydrophobic^37^ and electrostatic^38^ interactions and to determine domain organizations from the spontaneous reconstitution of native domains after mixing of multiple subdomain fragments^29–31^.

Here we have studied the role of the length of the loop connecting two EF-hand subdomains in ligand binding and stability. We have produced a series of calbindin D_9k_ (S100G) variants in which the linker between the two EF-hands is expanded by 1–16 glycine residues. We measured the effects on the free energy of Ca^2+^ biding using a chelator-based assay and the stability towards unfolding using circular dichroism spectroscopy. All measurements were performed in comparison with the wild-type proteins as well as a subdomain mixture of two fragments of the protein chain, each containing one EF-hand.

## Methods

### Molecular cloning

All mutants used in this study were based on the P43M mutant of the 75-residue protein bovine minor A calbindin D_9k_, which we henceforth refer to as “G0”. Insertions of 1 to 16 glycine residues between sites 43 and 44 in the calbindin D_9k_ gene were performed using PCR with a set of primers for stepwise introduction of glycine codons. The proteins were expressed in *E. coli*, purified to their Ca^2+^-free form as described^39^, and stored in lyophilized form until use. The purity was confirmed using SDS PAGE, agarose gel electrophoresis in Ca^2+^ and EDTA, NMR spectroscopy and Ca^2+^-titrations at elevated pH (8.1). The latter was used to rule out contamination by EDTA relying on the significantly enhanced Ca^2+^-affinity for EDTA but not calbindin at elevated pH^40^.

We use a nomenclature in which G0 represents the P43M mutant, G1 has one glycine inserted, and so on.

### Differential scanning calorimetry

Protein samples were dissolved in 10 mM sodium phosphate, 0.5 mM EDTA, pH 7.5 at concentrations of about 3 mg/ml (ca. 300 μM). For each mutant, four to six scans were performed at a rate of 60 °C/h in a range from 10 to 110 °C in a VP-DSC calorimeter (Microcal). Scans were performed alternatingly up and down. A reference scan of buffer without added protein was subtracted from each such obtained curve and the peak value determined using a two-state transition model with the built-in software. The protein concentrations were determined by UV absorbance spectroscopy using an extinction coefficient of 1490 M/cm.

### Urea denaturation

For each of the mutants, a set of samples with increasing urea denaturant concentration was obtained by mixing solutions of 20 μM protein in 10 mM sodium phosphate, 0.5 mM EDTA, pH 7.5 and 20 μM protein in 10 mM sodium phosphate, 0.5 mM EDTA, 9.75 M urea, pH 7.5. For each experiment, 41 samples were prepared in this way. The degree of denaturation was measured by circular dichroism spectroscopy in a 2 mm cuvette using a Jasco J-720 spectrometer at 20 °C. The signal at 222 nm was used as a measure of the amount of secondary structure (calbindin D_9k_ is mainly helical).

A simple two-state model^41,42^ was fitted to the data using Igor Pro:

$$\Delta {G}^{0}=\Delta {G}^{0}({\text{H}}_{2}{\text{O}})-m\left[{\text{urea}}\right]=-RT\,\mathrm{ln}\it{K=-RT}\,\mathrm{ln}\frac{\it{F}_{\text{app}}}{1-\it{F}_{\text{app}}},$$

where ${F}_{\text{app}}=\frac{y-{y}_{f}}{{y}_{u}-{y}_{f}}$ is the fraction of unfolded protein at a certain urea concentration and $y$ is the CD signal. The baselines before and after the transition area were considered linear and were introduced in the fit as ${y}_{f}={y}_{f}^{0}+{m}_{f}[{\text{urea}}]$ and ${y}_{u}={y}_{u}^{0}+{m}_{u}[{\text{urea}}]$. Combining these equations, we arrive at:

$$y=\frac{{y}_{f}^{0}+{m}_{f}\left[{\text{urea}}\right]+\left({y}_{u}^{0}+{m}_{u}\left[{\text{urea}}\right]\right)\mathrm{exp}\left(-\frac{1}{\it{RT}}\left(\Delta {\it{G}}\,^{0}({\text{H}}_{2}{\text{O}})-{m}\left[{\text{urea}}\right]\right) \right)}{1+\mathrm{exp}\left(-\frac{1}{\it{RT}}\left(\Delta {\it{G}}\,^{0}({\text{H}}_{2}{\text{O}})-{m}\left[{\text{urea}}\right]\right) \right)}.$$

The fitted parameters in this case are ${y}_{f}^{0},{y}_{u}^{0},{m}_{f}, {m}_{u}, \Delta {G}^{0}({\text{H}}_{2}{\text{O}}),m$.

### Ca^2+^ binding

Protein was dissolved at concentrations of about 20 μM in a Ca^2+^-free buffer (2 mM Tris pH 7.5) containing 25 μM of the Ca^2+^ chelator Quin-2, which changes its UV absorbance spectrum depending on the amount of bound Ca^2+^^26,43^, 5 μl aliquots of 2.654 mM CaCl_2_ were titrated stepwise into a sample of 2.5 ml. The competitive binding of Ca^2+^ to protein and chelator is reflected in the absorbance at 263 nm, which decreases as Quin-2 binds Ca^2+^, and was recorded at each titration step. The macroscopic binding constants were then estimated by fitting to the data an equation describing the competition for calcium ions between Quin-2 and the protein, assuming (verified experimentally) that absorbance changes arise due to Ca^2+^ binding to Quin-2, using the software CaLigator^44^. Titrations were performed in triplicate. Reported are the means and standard error of these measurements.

The stabilization due to Ca^2+^ binding we report here is defined as the free energy change upon Ca^2+^ binding assuming a standard state of 1 M and is given by $\Delta {G}^{0}=-RT\,\mathrm{ln}\left({\it{K}}_{1}{\it{K}}_{2}\right),$ with the binding constants in units of M^−1^.

A lower limit for the cooperativity is obtained for the case of equal values of the microscopic binding site affinities *K*_I_ and *K*_II_ in terms of ${\Delta \Delta G}_{\text{min}}=-RT\,\mathrm{ln}(4{\it{K}}_{2}/{\it{K}}_{1})$^41^. This is a lower bound for the binding cooperativity, which is higher (more negative ΔΔ*G*) if the two microscopic binding sites are different.

### Polymer linker model

To extrapolate the energetic influence of linker length we assumed that the inserted glycine residues can be described as an unstructured polymer chain. We further allowed the existence of a pre-existing loosely structured linker, even in the G0 variant^8^. In this model, the thermodynamic stability can be approximated by^5,8^ $-\Delta {G}^{0}\left({\text{H}}_{2}{\text{O}}\right)=-\Delta {G}_{\text{ref}}^{0}+cRT\,\mathrm{ln}\left(\frac{\it{L}+{\it{L}}_{\text{off}}}{{\it{L}}_{\text{ref}}+{\it{L}}_{\text{off}}}\right)$, where we set the reference length ${L}_{\text{ref}}=16$. The parameter $c$ describes the type of polymer where $c=1.5$ represents an ideal random-walking chain and $c=1.63$ describes excluded volume effects^5^. We also introduced ${L}_{\text{off}}$ to describe the length of any pre-existing linker^8^. In our data, both $c=1.5$ and $c=1.63$ fitted the data equally well and resulted in a fitted pre-existing linker length ${L}_{\text{off}}$ of about 5 amino acids.

## Results

**Figure 1:**
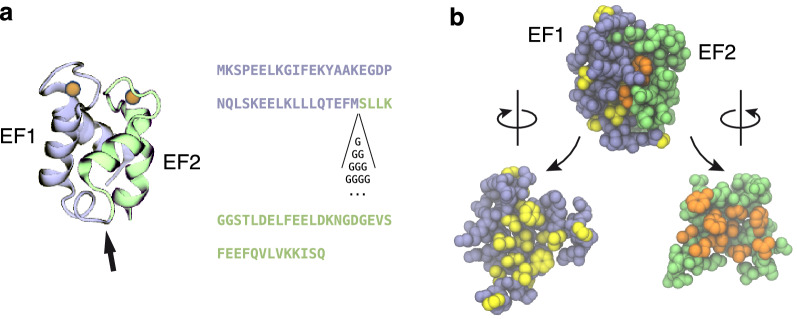
Structure of calbindin D_9k_. (**a**) Crystal structure and sequence of bovine calbindin D_9k_-P43M. Ca^2+^ ions are shown in orange. In our experiments, we inserted poly-glycine linkers of 1–16 residues into the linker region between the EF hands (arrow). (**b**) Backbone and hydrophilic side-chains are in purple and green and hydrophobic side chains in orange and yellow. The intact protein is shown on top. At the bottom the protein is artificially cut in two fragments between residues 43 and 44, the two parts separated and rotated 90° in opposite directions to reveal the inter-subdomain interface towards the viewer.

Our parent protein, G0, is equal to the bovine minor A from of calbindin D_9k_ (S100G) with the mutation P43M, which avoids heterogeneity due to cis–trans isomerization of Pro43^45,46^. At the same time, the mutation introduces a methionine residue allowing CNBr cleavage to be used for EF-hand fragment production^16^. The mutants carry insertions of 1–16 glycine residues between Met43 and Ser44, i.e. in the linker region between the two subdomain EF-hands (Fig. 1).

### Thermodynamic stability as a function of linker length between EF hands

**Figure 2:**
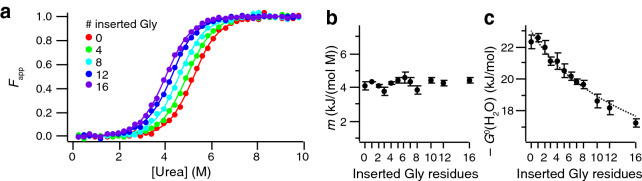
Urea denaturation as a function of linker length in the absence of Ca^2+^. (**a**) Examples for urea denaturation curves of apo calbindin D_9k_ with different linker lengths. Folding was assessed by monitoring the helicity of the protein as reported by circular dichroism at 222 nm (filled circles). The data could be well fit with a two-state folding model (continuous lines). (**b**) The *m*-values for the transition region of the denaturation curves. (**c**) Thermodynamic stability as a function of linker length assuming a fixed *m*-value of 4.28 kJ/(mol M). Dashed line: fit to an entropy-based destabilization model (see “Methods”: Polymer linker model, $c=1.63$) with fitted values $-\Delta {G}_{\text{ref}}^{0}=17.5\pm 0.1 \text{kJ/mol}$ and ${L}_{\text{off}}=5.6\pm 0.6$. Values and error bars in (b) and (c) are based on weighted averages from two independent experiments.

In a first set of experiments we determined the destabilization due to the insertion of a variable length poly-glycine linker between the EF hands. To this end, we monitored the far-UV circular dichroism (CD) signal at 222 nm, which is mainly used as a reporter for helicity in folded polypeptides, as it changes upon increasing the concentration of urea in the buffer from 0 to 9.75 M (Fig. 2a). The signal shows a clearly defined transition from folded (low values) to unfolded (high values) at around 5 M urea with small but distinct variation over the variants. The data for each mutant could be well fitted by a two-state folding model (continuous lines in Fig. 2a). The midpoint of the transition shifts in a systematic manner to lower concentrations of denaturant as the linker length is increased. In contrast, the *m*-values appear to be independent of linker length (Pearson’s *r* = 0.22 ± 0.31, slope = 0.002 ± 0.009, Fig. 2b) and average at 4.28 ± 0.03 kJ/(mol M). Denaturation studies of a large number of proteins have suggested that the *m*-value correlates with the amount of protein surface exposed to solvent upon unfolding^47^. Since this property is not altered by Gly-linker-extension, it is rewarding to find the nearly constant *m*-values over our variant series. The consistent *m*-values justify a comparison of the extrapolated free energy of folding of the mutants in the absence of denaturant, Δ*G*^0^(H_2_O) (Fig. 2c). The near-linear relationship between Δ*G*^0^(H_2_O) and linker length implies that the stability towards unfolding decreases in a systematic manner upon extending the linker between the EF-hand subdomains. Destabilization predictions based on polymer lattice models^48^ agree well with the experimental data (see methods: polymer linker model and dashed line in Fig. 2c).

**Figure 3:**
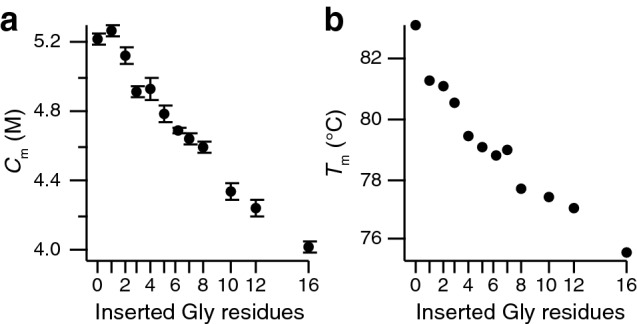
Thermodynamic stability of apo calbindin D_9k_ as a function of linker length between the EF hands. (**a**) Midpoint of urea denaturation. (**b**) Melting point as determined by DSC.

Further insight comes from thermal denaturation studies, where we measured the thermal melting for each of the mutants using differential scanning calorimetry (DSC). The melting temperature decreases from 83 °C for G0 to 76 °C for G16. In agreement with chemical denaturation (Fig. 3a), also DSC (Fig. 3b) corroborates the destabilization of calbindin D_9k_ by insertion of the linker and reveals a monotonic decay in *T*_m_ with linker length.

It is important to note that the denaturation processes only go to completion for Ca^2+^-free calbindin D_9k_ within the ranges studied (up to 9.75 M urea and 110 °C, respectively). Ca^2+^ binding increases the stability significantly and pushes the transition region outside of the accessible range^49^. Indeed, the high thermal stability of calbindin D_9k_ allows boiling to be used early in the purification protocol to selectively precipitate *E. coli* proteins.

### Ca^2+^ affinity as a function of linker length between EF hands

**Figure 4:**
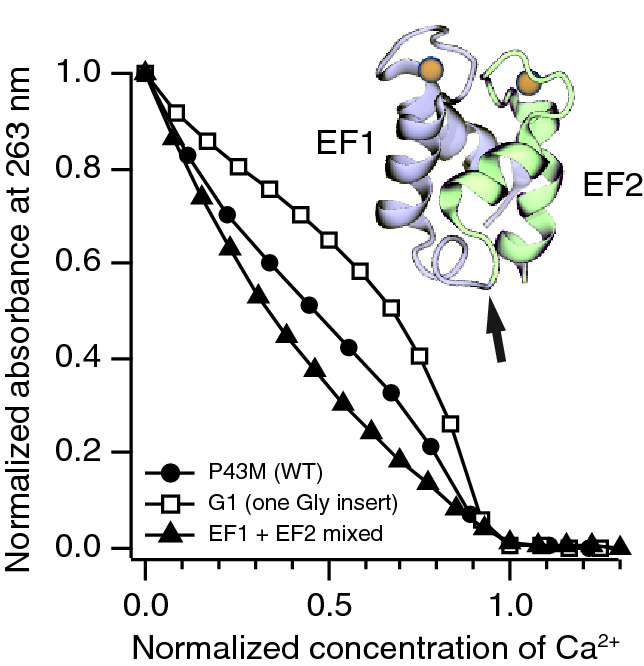
Ca^2+^ binding assay for mutants of calbindin D_9k_. Titrations with CaCl_2_ are performed in the presence of Quin-2, a competing Ca^2+^-chelating agent that changes its absorbance at a characteristic wavelength of 263 nm. S-shaped curves indicate cooperativity in Ca^2+^ binding. The curves are scaled such that they coincide at a normalized Ca^2+^ concentration of 1.0, relative to the concentration of available binding sites (i.e. the Quin-2 concentration plus two times the protein concentration). Inset: Structure of calbindin D_9k_ with bound calcium ions (orange). The arrow indicates the site where poly-glycine linkers were inserted.

In another set of experiments, we asked how the Ca^2+^-binding properties, and especially binding cooperativity, are affected by the insertion of the linker. To this end, we performed Ca^2+^ titrations on each of the mutants in the presence of a competing Ca^2+^-binding chelator, Quin-2, which changes its UV absorbance at 263 nm upon Ca^2+^ binding^50^ (Fig. 4). An S-shaped curve is observed when *K*_2_ > *K*_1_/4, i.e. in the limit of positive cooperativity. A curve with a single bend is seen when *K*_2_ = *K*_1_/4, which occurs when the two sites have equal affinity and no cooperativity, or when cooperativity is masked by different affinities for the two sites. Widely different affinities leading to sequential binding appear as an opposite S-shape. In the present case, we observed the S-shape indicative of positive cooperativity for all variants G0-G16. We then proceeded to fit the measured values to a competitive binding model using the software CaLigator^44^. In the case of the P43M mutant with no glycine inserted (“G0”), we obtained lg *K*_1_ = 7.91 ± 0.03 and lg *K*_2_ = 8.61 ± 0.03, which is close to the previously measured values of 7.75 ± 0.04 and 8.59 ± 0.04, respectively^28^.

**Figure 5:**
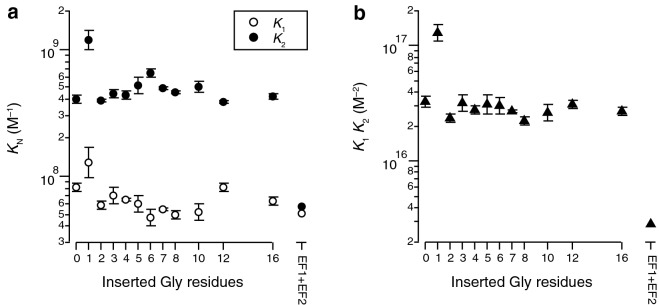
Macroscopic Ca^2+^ affinities to calbindin D_9k_ from titrations in the presence of Quin-2. (**a**) Individual values. (**b**) As a matter of the technique, the product *K*_1_*K*_2_ is better defined than the individual values. Error bars are based on weighted averages from experiments in triplicate.

The macroscopic Ca^2+^-binding constants as a function of the number of inserted glycines are shown in Fig. 5. Overall, there is no measurable general influence of linker length on the values of *K*_1_ or *K*_2_ (lg *K*_1_: *r* = − 0.14 ± 0.33, slope = − 0.004 ± 0.002; lg *K*_2_: *r* = − 0.01 ± 0.33, slope = − 0.001 ± 0.001; Fig. 5a). Interestingly, the sole exception to this rule is the mutant G1 with one glycine inserted between residues 43 and 44. Both lg *K*_1_ (8.11) and lg *K*_2_ (9.07) are significantly higher than the values for all other investigated mutants (7.79 ± 0.08 and 8.66 ± 0.06, respectively). Also the product lg *K*_1_*K*_2_ (17.11), which is better defined than the individual macroscopic constants due to correlation of the fitting parameters, is independent of linker length for all mutants except G1 (Pearson’s *r* = − 0.23 ± 0.32, slope = 0.000 ± 0.003) and higher for G1 than for all other mutants (lg *K*_1_*K*_2_ = 16.45 ± 0.05, Fig. 5b).

**Figure 6:**
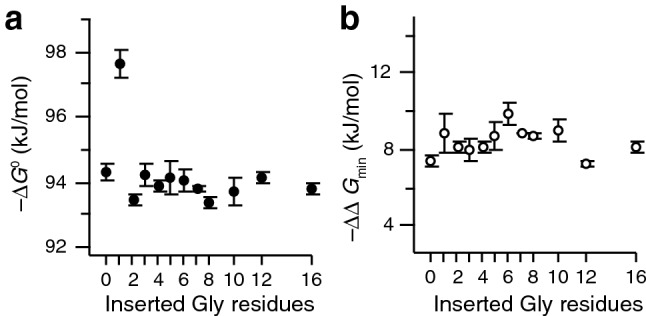
Energy coupling of ligand binding and folding. (**a**) Stabilization due to the binding of Ca^2+^. (**b**) Lower bound for the free energy coupling between binding events, i.e. the cooperativity of Ca^2+^ binding. Error bars are standard errors from triplicate experiments.

Two measures allow assessing the thermodynamic effects of Ca^2+^ binding. The first measure is the free energy of Ca^2+^ binding. We report here the quantity $\Delta {G}^{0}=-RT\,\mathrm{ln}\left({\it{K}}_{1}{\it{K}}_{2}\right)$, valid at the standard state of 1 M. This quantity naturally follows the same trend as *K*_1_*K*_2_, with G1 as the only exception to an otherwise linker-length independent trend (Fig. 6a).

The second quantity, $\Delta \Delta {G}_{\text{min}}$, is a limiting value for the cooperativity between the two calcium ions, i.e. the difference in free energy between binding of the second and the first ion. When $\Delta \Delta {G}_{\text{min}}$ is negative, the binding occurs with positive cooperativity. The values for $\Delta \Delta {G}_{\text{min}}$ as a function of linker length are shown in Fig. 6b. There is no significant dependence of cooperativity on the number of inserted glycines between the EF hands. Interestingly, also the previously identified outlier G1, which showed elevated stabilization due to Ca^2+^ binding, follows this trend. We find an average value of − 8.6 ± 0.1 kJ/mol for the cooperativity, independent of linker length (*r* = 0.00 ± 0.28, slope = 0.032 ± 0.020).

**Figure 7:**
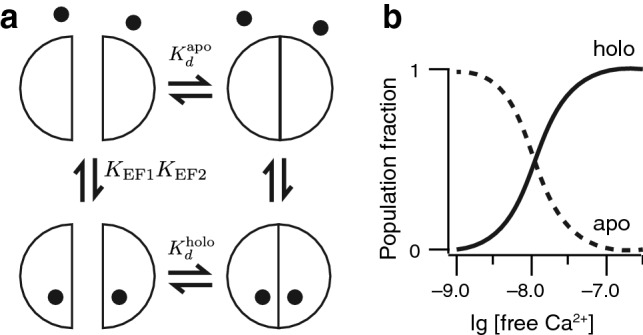
Thermodynamics of EF hand association. (**a**) Thermodynamic scheme for Ca^2+^ titrations to EF1 + EF2. The values ${K}_{\text{EF1}}{K}_{\text{EF2}}$ and ${K}_{d}^{\text{holo}}$ are known from literature^34,37^. The two top states represent apo conformations, the two lower states holo conformations. States with only one calcium ion bound are left out for simplicity. (**b**) Calculated populations of the aggregate holo conformations and apo conformations for an assumed ${K}_{d}^{\text{apo}}$ of 100 μM.

Knowing that calbindin D_9k_ can be reconstituted from its EF hand fragments^34,37^, we repeated the titration study starting with equimolar concentrations of Ca^2+^-free EF1 and EF2, at the same concentration as in all other experiments. The data showed that this pair has relatively high apparent Ca^2+^ affinity (lg *K*_1_ = 7.70, lg *K*_2_ = 7.76, Figs. 4, 5), yet on average a factor of 3 lower than the intact protein. Because of the coupling between fragment assembly and Ca^2+^ binding, this measured affinity depends on the fragment concentration and is hence an apparent affinity (Fig. 7). From the ratio of *K*_2_ and *K*_1_ we obtain a lower limit to the cooperativity of $\Delta \Delta {G}_{\text{min}}$ = –3.8 kJ/mol, compared to an average value of − 8.6 ± 0.1 kJ/mol for G0–G16 (see above). This could either mean that the cooperativity in the case of separate fragments is indeed lower, or that the Ca^2+^ affinity for the two sites is more different when they occur on separate polypeptides. For intact calbindin D_9k_ it has been estimated based on NMR spectroscopy that the two sites have equal affinities with a ratio between 1 and 3^41^, meaning that ΔΔ*G* is between − 8.6 and − 9.3 kJ/mol. For the separate EF-hands, the affinities for the two sites are estimated to differ by a factor of 5, in which case ΔΔ*G* would be − 5.2 kJ/mol. This suggests that in addition to effects caused by differences in the individual site affinities, the cooperativity in the case of separate EF-hands is indeed reduced.

## Discussion

Here we have studied the influence of covalent backbone linkage and linker length between two EF-hand subdomains of the small Ca^2+^ binding protein calbindin D_9k_. ID="Par26">Previous studies have shown that—like other Ca^2+^ binding proteins such as troponin C^15^—calbindin D_9k_ can be readily reconstituted from EF hand fragments^16,34,37^. Reconstitution experiments using surface plasmon resonance have revealed a high affinity (${K}_{D}^{\text{holo}}\approx 3 {\,\text{pM}}$) between the EF hands when Ca^2+^ was present in the sample buffer^37^. Each monomeric EF hand in isolation has a Ca^2+^ affinity of around $\mathrm{lg}K\approx 4.4-4.6$^34^. The free energy of folding of P43M under Ca^2+^-free conditions (≈ 22.6 kJ/mol) is an upper limit for the absolute free energy involved in the dimerization of EF1 and EF2. We can hence infer an upper limit for the ${K}_{D}^{\text{apo}}$ of dimerization to be  ≈ 100 μM. This value coincides with the homo-dimerization constant of Ca^2+^-free EF1, of around $\mathrm{lg}K\approx 4$^34^. At the protein concentrations used in our Ca^2+^ titration (≈ 25 μM), the two EF hands are therefore likely to be monomeric at very low Ca^2+^ concentrations. The three orders of magnitude higher apparent Ca^2+^ affinities for the EF1-EF2 mixture $(\mathrm{lg}{K}_{1}=7.70$ and $\mathrm{lg}{K}_{2}=7.76$) compared to the monomeric isolated EF hands may be reconciled in terms of the thermodynamic linkage of Ca^2+^ binding and hetero-dimer formation. In the description of a thermodynamic scheme such as shown in Fig. 7, we measure the shift from the two upper states to the two lower states in our Ca^2+^ titration assay. The values for ${K}_{{\text{E}}{\text{F1}}}{K}_{\text{EF2}}$ and ${K}_{d}^{\text{holo}}$ are known from literature^34,37^. For an assumed affinity between apo-EF1 and apo-EF2 of ${K}_{D}^{\text{apo}}$ = 100 μM, the equilibrium is established mainly via the left half of the scheme in Fig. 7, i.e. Ca^2+^ association to the individual EF hands is followed by subdomain association. We calculate the transition between the two upper apo-states and the two lower holo-states to be at about 10^–7.9^ M Ca^2+^, close to the measured values of lg *K*_1_ and lg *K*_2_. The measured Ca^2+^ affinities for the mixture of EF1 and EF2 are hence rather an effect of the high affinity between the Ca^2+^-bound EF-hands than an effect of high individual Ca^2+^ affinities.

It is expected that in the limit of very long linker lengths the Ca^2+^ binding data converge to the case of unconnected EF hands, where binding cooperativity is almost abolished. However, our data revealed that up to a size of 16 inserted glycines there is no adverse effect on the binding cooperativity of Ca^2+^. This prompts the question what the minimal linker length is to observe a significant drop in Ca^2+^ binding cooperativity. Assuming that the connecting linker between EF1 and EF2 acts mainly as a mechanical tether that keeps the EF hands in spatial proximity, we can use polymer models to estimate the effective concentration of EF1 and EF2 as a function of linker length^51^. In the case of a G16 linker, we obtain an effective concentration of ≈ 130 mM, much larger than our previously estimated ${K}_{D}^{\text{apo}}$. In the picture of Fig. 7, the insertion of a linker hence keeps the local effective concentration of EF1 and EF2 high enough to force EF1 and EF2 to form an intra-molecular complex, which is equivalent to them forming a reconstituted domain. Using the same model, we estimate that several hundreds of amino acids are needed in the linker to significantly shift this equilibrium.

Here the two EF-hand fragments were connected via a largely unstructured poly-Gly linker of variable size. Over the whole series and even in the limit of the 16-residue linker there was little effect on the overall Ca^2+^ affinity or cooperativity. The only exception was the mutant G1 with one inserted glycine, which displayed a higher overall affinity for Ca^2+^, yet unaltered binding cooperativity. The site we chose for linker insertion is in the linker region between EF1 and EF2, far away from the Ca^2+^-binding sites (see Fig. 1). We hypothesize that the increase in Ca^2+^ affinity is due to favorable rearrangements of the interface between EF1 and EF2 in the holo protein, made possible by the insertion of an additional residue. This model was tested in MD simulations; however, no rearrangements were observed over 100 ns trajectories (see Supplementary Text and Fig. S1). The detailed reasons for the difference of the G1 variant therefore warrant further investigation in future work.

While Ca^2+^ binding was generally unaffected, we observed a significant destabilization of the protein upon increasing the linker length. A recent study on the linker length effects in other proteins have shown that this effect is conveyed by entropic destabilization^11^ and was also observed in other proteins, such as the four-helix bundle Rop^5,6^, the chymotrypsin inhibitor-2^7^, yeast phosphoglycerate kinase^9^—a monomeric two-domain protein used as folding model representative of large proteins—or cytochrome c^10^. This destabilization is of entropic nature and described in polymer theory^48^. In the case of Ca^2+^-free calbindin D_9k_ we observed a destabilization between G0 and G16 by 23%. We extrapolate that many hundreds inserted residues are needed to obtain an equal population of folded and unfolded states under non-denaturing conditions at room temperature and zero Ca^2+^. Notably, our measured destabilization of G10 compared to G2 of $\mathrm{\Delta \Delta }{G}^{0}=2.3\pm 0.2$ kJ/mol is compatible with the reported destabilization in ⍺ spectrin SH3 (≈ 2.8–3.5 kJ/mol)^8^ but differs from reports for Rop (≈ 10.5 kJ/mol)^5^, suggesting that, in addition to entropic effects from unstructured polymer chains, additional factors may modulate the energetic impact of loop length variations. In addition to the thermodynamic impact, which we focused on in our work, variations of the linker length may also have a kinetic impact. Variation of linker-induced drag may affect the motion coupling between the EF hands and consequently kinetically influence the coupled binding/folding. Walsh et al. have highlighted this effect in NMR studies of the coupled motion between connected domains of GB1^52^.

The native stability of the two separated subdomains can also be rescued by restoring an alternative covalent linkage between subdomains. This has been shown for a calbindin D_9k_ variant in which two cysteines were substituted for residues 39 and 73, whose side chains were located in the native structure in optimal positions for disulfide bonding^28^. The stability is highly similar in native (43–44-linked) and reconstituted disulfide linked (39–73 linked) calbindin D_9k_^28^. A doubly linked protein (43–44 and 39–73 linkages) is significantly more stable^28^.

Previous work has shown that the G0 variant of calbindin D_9k_ can slowly assemble into an EF-hand-swapped dimeric configuration on the time-scale of days and weeks^17,20^. This process of oligomerization is a feature of a wider range of proteins^53^, and, if unchecked, may lead to the formation of protein gels in the form of extended networks^22^ or fibrils through run-away domain swapping^21^. In the case of calbindin D_9k_, domain swapping is accelerated by the P43M mutation present in all our variants^20^. All variants of the current study remained monomeric at the concentrations (20–300 µM), buffer conditions (pH 7.5, low ionic strength, no or low Ca^2+^ concentration) and time scale (minutes to hours) of the current work (Fig. S2). Still, it is intriguing to speculate whether the additional introduction of a long linker between the EF hands facilitates domain swapping. We tested this by incubation of samples at much higher protein concentration (2.5 mM), higher ionic strength, in the presence of Ca^2+^, at lower pH (5.0 and 6.0) for prolonged time (Fig. S3). Although the monomer is still by far the dominant form of all variants after 48 days, it is evident that several variants can form a range of oligomers, with up to seven oligomeric forms for G12, albeit under sample conditions very far from those of the current denaturation and Ca^2+^ binding studies.

## Conclusions

The findings in this work highlight the significance of covalency in proteins. While proteins can be reconstituted from fragments and these reconstituted proteins can also show enzymatic activity^13^, we demonstrated here that for calbindin D_9k_ a covalent connection between the two EF hand fragments is necessary to achieve native Ca^2+^-binding affinity and cooperativity. This connection can consist of an unstructured flexible linker of up to 16 glycine residues—possibly even far more—that must only provide floppy mechanical linkage for the protein to retain its native properties. However, if the connection is broken, as in a fully reconstituted system EF1 + EF2, the Ca^2+^-binding affinity as well as cooperativity between the two binding sites is reduced.

Our findings point to a possible pathway for the evolutionary optimization of ligand-binding proteins. Apparently, there is considerable evolutionary freedom to insert unstructured residues between structured regions of proteins without influencing their ligand binding properties. Nature may here have a way to fine-tune a protein's thermodynamic stability and concomitantly, also its degradability, while leaving its function, the sequestration of ligands, untouched. In our model system calbindin D_9k_, there is only little structural rearrangement upon Ca^2+^ binding. However, it is conceivable that in other proteins where significant conformational rearrangements occur upon ligand binding, evolutionary variation of linker lengths may also alter the cooperativity of binding enabling control over the response to small variations ligand concentration without perturbing the overall affinity.

## Supplementary information

Supplementary Information.

## Data Availability

All data generated or analyzed during this study are included in this article. MD trajectories are available upon request.
