# Supplementary material for: Benefits and constrains of covalency: the role of loop length in protein stability and ligand binding
Source: Sci Rep. 2020 Nov 18;10:20108. doi: 10.1038/s41598-020-76598-x (PMC7674454; doi:10.1038/s41598-020-76598-x)
Supplement: Supplementary file 1 — Supplementary Information. [file 41598_2020_76598_MOESM1_ESM.pdf]

Supplementary Information for

Benefits and constrains of covalency: the role of loop length in protein stability and ligand binding

Sara Linse[1*], Eva Thulin[1], Hanna Nilsson[1] and Johannes Stigler[1,2*]

[1]Departments of Biophysical Chemistry, Biochemistry and Structural Biology, Lund University Sweden

[2]Gene Center, Ludwig-Maximilians-University, 81377 Munich, Germany

[*]Address correspondence to Johannes Stigler (stigler@genzentrum.lmu.de) or Sara Linse (sara.linse@biochemistry.lu.se)

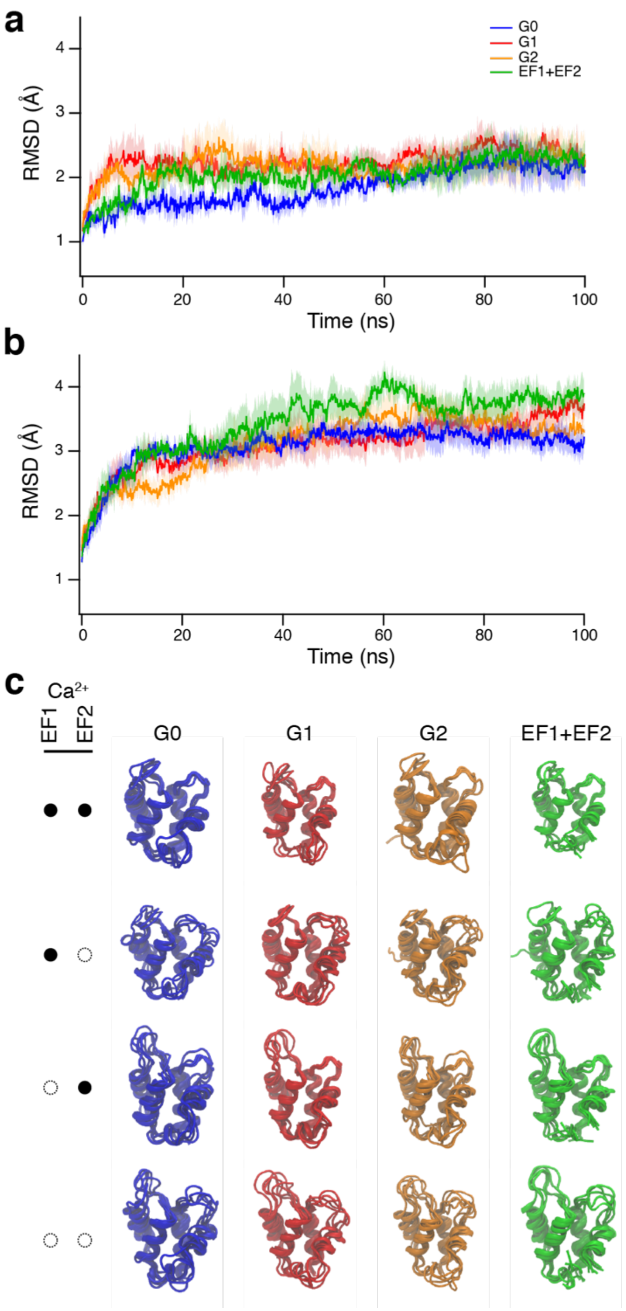

Figure S1. All-atom molecular dynamics (MD) simulation of protein variants in different Ca^{2+}-bound states. **(a)** Average RMSD difference to 4ICB of five replicate simulations each, with Ca^{2+} bound to both EF1 and EF2. **(b)** Average RMSD difference to 4ICB of five replicate simulations each, without Ca^{2+}. **(c)** Average structures of the last 20 ns of each MD run. Each structure is an overlay of 5 replicates. Ca^{2+} ligands are not shown.

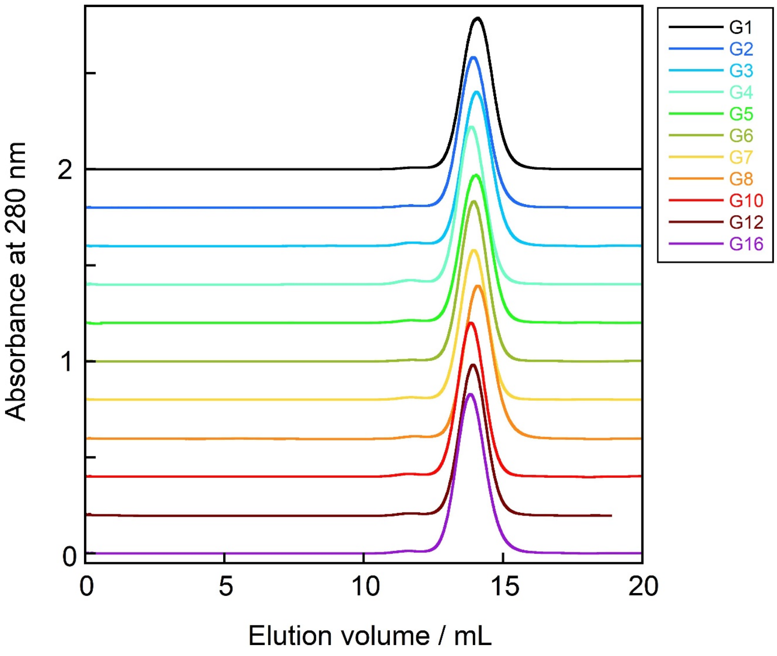

Figure S2. Test for oligomerization and domain swapping under standard conditions. Each variant was dissolved from lyophilized powder at 3 mg/mL (ca. 300 μM) in 10 mM sodium phosphate buffer with 0.5 mM EDTA, pH 7.5 and directly analyzed using size exclusion chromatography in the same buffer on a Superdex 75 column (GE Healthcare). All chromatograms except for G16 have been y-translated.

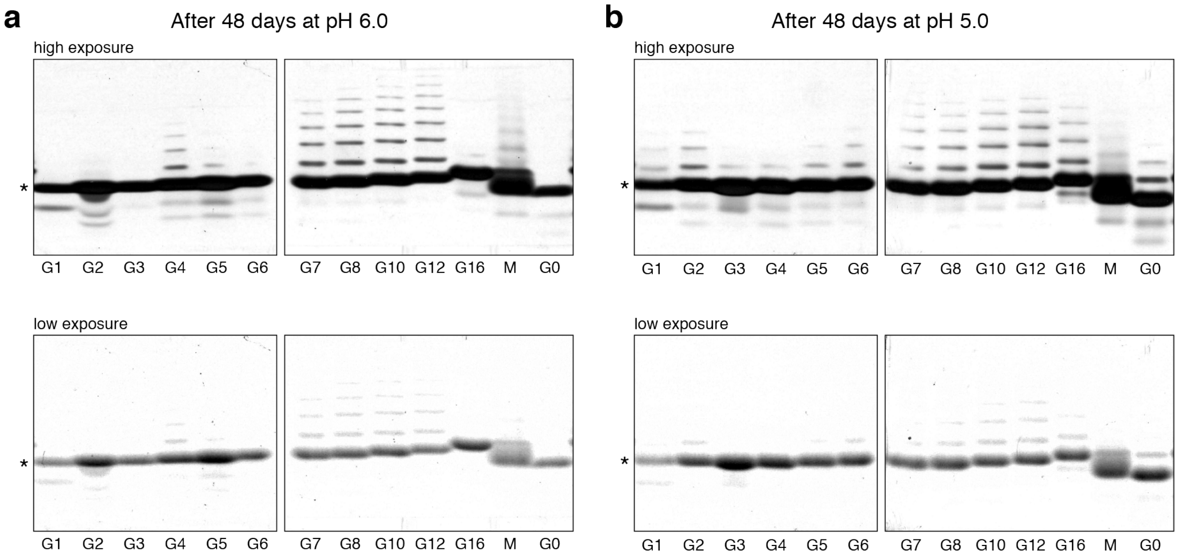

Figure S3. Long-term test for oligomerization or domain swapping. Each variant was dissolved at 2.5 mM in **(a)** 20 mM MES, 100 mM NaCl, 4 mM $CaCl_2$, 0.02% NaN_3, pH 6.0, or **(b)** 20 mM NaAc, 100 mM NaCl, 4 mM $CaCl_2$, 0.02% NaN_3, pH 5.0, and incubated for 48 days followed by separation on native PAGE (15% gel) and staining with Coomassie Brilliant Blue. The calcium concentration (4 mM) was chosen below saturation (5 mM) to facilitate swapping through co-existence and exchange between Ca2+-bound and -free forms.[1] The lane marked M shows the results for a mix of all 12 variants in equimolar proportions, which had been incubated for 48 days. Asterisks mark approximate positions of monomeric proteins. Other bands are oligomeric forms.

Supplementary Text

To investigate if major structural rearrangements of the interface between EF1 and EF2 result in the observed increased calcium affinity of the mutant G1 we performed all-atom MD simulations (see Supplementary Methods). We created a series of mutants (G0, G1, G2 and a mixture of EF1+EF2) in all possible ligation states, i.e. in apo, with Ca^{2+} bound to EF1, EF2 or both, based on the PDB structure 4ICB. After relaxation, we performed equilibrium trajectory simulations in explicit solvent at 300 K for 100 ns with five replicates for each configuration. The trajectories appeared to approach an equilibrium state after about 40 ns (**Fig. S1a,b**). We then calculated average structures of the last 20 ns of each run and aligned them to a common template (**Fig. S1c**). While there were differences between runs, no significant rearrangements of the interface between EF1 and EF2 were found, suggesting that rearrangements, if they occur, take place on time scales larger than the available simulation time of 100 ns.

Supplementary Methods

Molecular dynamics simulations

The structures for all-atom molecular dynamics (MD) simulations were based on the PDB structure 4ICB.[2] The P43M and glycine insertion mutations were created in VMD.[3] Simulations were performed in explicit solvent with 2 fs time steps using the CHARMM36 force field as implemented in NAMD.[4] Structures underwent an energy minimization run for 500 steps, followed by an equilibration run of 100 ns at 300 K. Five replicates were performed for each configuration.

Supplementary References

1. Håkansson, M., Svensson, L. A., Fast, J. & Linse, S. An extended hydrophobic core induces EF-hand swapping. *Protein Sci* **10,** 927–933 (2001).
2. Svensson, L. A., Thulin, E. & Forsén, S. Proline cis-trans isomers in calbindin D9k observed by X-ray crystallography. **223,** 601–606 (1992).
3. Humphrey, W., Dalke, A. & Schulten, K. VMD: visual molecular dynamics. *J Mol Graph* **14,** 33–8– 27–8 (1996).
4. Phillips, J. C. *et al.* Scalable molecular dynamics with NAMD. *J. Comput. Chem.* **26,** 1781–1802 (2005).
